# Establishment of a chronic aspiration pneumonia mouse model using oropharyngeal aspiration of food suspension

**DOI:** 10.1002/ame2.70197

**Published:** 2026-03-24

**Authors:** Qianwen Li, Fansen Lin, Hongzhi Gao, Yun Zhao, Yuqi Liu

**Affiliations:** ^1^ Department of Critical Care Medicine The Second Affiliated Hospital of Fujian Medical University Quanzhou Fujian China; ^2^ Fujian Key Laboratory of Lung Stem Cells The Second Affiliated Hospital of Fujian Medical University Quanzhou Fujian China; ^3^ Department of Neurosurgery The Second Affiliated Hospital of Fujian Medical University Quanzhou Fujian China; ^4^ Department of General Surgery, Nanjing BenQ Medical Center The Affiliated BenQ Hospital of Nanjing Medical University Nanjing Jiangsu China; ^5^ Department of Surgical Research Laboratory, BenQ Medical Center The Affiliated BenQ Hospital of Nanjing Medical University, The Clinical Translational Research Center for Surgical Infection and Immunity of Nanjing Medical University Nanjing Jiangsu China; ^6^ Fujian Provincial Center for Respiratory Medicine Quanzhou Fujian China

**Keywords:** aspiration pneumonia, disease models, lung injury, pneumonia

## Abstract

**Background:**

Aspiration pneumonia (AP) is a prevalent and life‐threatening pulmonary disease resulting from repeated aspiration of exogenous materials such as food or gastric contents. However, most existing animal models simulate only acute injury and fail to reproduce the chronic pathological features observed in patients. This study aimed to establish a stable and clinically relevant chronic AP mouse model.

**Methods:**

First, four inhalation techniques including unilateral vagotomy (UV), intranasal inoculation (IN), intratracheal instillation (IT), and oropharyngeal aspiration (OA) were evaluated for model establishment in mice. The optimal method OA was applied to administer normal saline (NS), food suspension (FS), or gastric contents (GC) once weekly. Disease progression was evaluated at 2, 4, and 8 weeks. Lung injury was evaluated by tracer distribution, histopathology, cytokine profiling, transcriptomic analysis, and micro‐computed tomography (micro‐CT).

**Results:**

OA achieved stable pulmonary delivery. Both FS and GC induced lung injury. However, 8‐week FS exposure resulted in more severe and persistent damage, characterized by increased alveolar–capillary barrier permeability and elevated cytokine and chemokine levels. Histology showed FS‐specific obstructive bronchiolitis with retained food particles, closely mimicking human aspiration pneumonia. Micro‐CT revealed gravity‐dependent pulmonary consolidation, most evident in the FS group. Transcriptomic analysis indicated activation of neutrophil extracellular traps (NETs) formation during chronic AP progression.

**Conclusions:**

This OA‐based FS model reproduced the key pathological and imaging features of human chronic AP and implicated NETs in the disease progression. It provides a robust platform for mechanistic investigation and therapeutic development.

## Graphical abstract

Four aspiration methods (UV, IN, IT, and OA) were compared, and OA was identified as the most effective delivery technique. A chronic aspiration pneumonia model was then established through repeated OA of food suspension or gastric contents. Integrated analyses, including micro‐CT, histopathology, cytokine profiling, and RNA sequencing, revealed progressive lung injury and pathological and imaging features closely resembling human aspiration pneumonia.

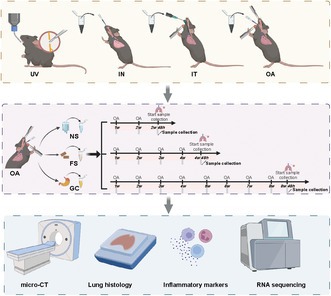

## 1 INTRODUCTION

Aspiration pneumonia (AP) is an inflammatory lung disease caused by the entry of exogenous materials such as food particles, gastric contents, or saliva into the lower respiratory tract.^1^ It most commonly affects individuals with dysphagia, altered mental status, or impaired airway protection mechanisms,^2^, ^3^ and is particularly prevalent among older adults and patients with neurological disorders.^4^, ^5^ Among hospitalized patients with pneumonia, the prevalence of AP has been reported to reach 66.8%,^6^ with an in‐hospital mortality rate as high as 21%, substantially exceeding that of non‐AP pneumonia.^7^, ^8^, ^9^, ^10^ Most cases involve recurrent, low volume occult aspiration,^11^, ^12^, ^13^ and dysphagia may represent an important pathogenic contributor.^3^ This chronic and often clinically silent process may go unrecognized, contributing to underdiagnosis and limiting opportunities for early intervention.^14^, ^15^, ^16^ Therefore, developing animal models that faithfully recapitulate the mechanisms of chronic aspiration is essential for understanding disease pathophysiology, identifying therapeutic targets, and developing effective interventions.

Most current experimental models focus on acute injury induced by a single aspiration event. Typical inhalants such as lipopolysaccharide (LPS) and hydrochloric acid are commonly used to model bacteria associated inflammation and chemical injury, respectively.^17^, ^18^, ^19^, ^20^ Differences in aspirate composition can drive distinct pathological trajectories.^21^ However, acute models based on single or short term exposures do not capture the sustained inflammation, barrier dysfunction, and tissue remodeling that characterize chronic, low dose aspiration. Commonly used aspiration methods, including intratracheal instillation (IT)^17^, ^20^ and unilateral vagotomy (UV),^19^ are invasive, technically demanding, and unsuitable for long‐term studies. Furthermore, they diverge from clinical conditions in both aspirate composition and delivery routes. Most use liquid aspirates, overlooking solid particle retention and granulomatous reactions, and rely on artificial injection rather than the natural aspiration process. Thus, there is a pressing need for a chronic AP model that better reflects clinical features, including aspirate composition, delivery pathway, physiological responses, and pathological progression.

Oropharyngeal aspiration (OA) is a noninvasive technique that employs the animal's natural inspiratory reflex to deliver material into the lungs.^22^ It causes minimal trauma, allows repeated application, and closely resembles the natural aspiration process in humans, making it particularly suitable for chronic models. Although OA has been used in several pulmonary disease models,^23^, ^24^, ^25^ it has rarely been adopted as an aspiration‐like delivery route to develop and systematically evaluate animal models of AP. Clinically, the most common aspirates are gastric contents (GC) and food residues,^1^, ^26^, ^27^, ^28^ the latter of which can be simulated by food suspension (FS). Previous chronic aspiration models have also used FS or GC, but typically administered these materials via IT.^29^, ^30^ While IT enables relatively controlled pulmonary delivery, its invasiveness may introduce procedure‐induced airway irritation that is unrelated to aspiration. This irritation may confound outcomes and limits its suitability for modeling long‐term, repeated exposure and physiologically relevant aspiration events.

In this study, we established a murine model of chronic AP using OA with either FS or GC. We systematically compared the effects of these two aspirates at weeks 2, 4, and 8. The evaluation focused on barrier integrity, inflammatory cell infiltration, cytokine responses, histopathological changes, imaging findings, and molecular mechanisms. By week 8, FS more closely reproduced the key pathological and radiological features of human chronic AP. It also revealed a potential role for neutrophil extracellular traps (NETs) in disease progression. This model provides a robust experimental platform for investigating the mechanisms of chronic AP and for developing targeted therapeutic strategies.

## 2 METHODS

### 2.1 Animals and experimental groups

Specific pathogen‐free (SPF) male C57BL/6 mice (6–8 weeks old, 22–26 g) were purchased from Jiangsu Huachuang Xinnuo Pharmaceutical Technology Co., Ltd. (license number: SYXK [Su] 2020‐0041). Mice were acclimatized for 7 days with free access to food and water under controlled conditions (20 ± 2°C, 50% ± 5% humidity, 12‐h light/dark cycle). All procedures were approved by the Institutional Animal Care and Use Committee of Fujian Medical University (IACUC FJMU 2025‐Y‐0346).

To establish a chronic aspiration pneumonia model, mice were exposed to different aspirates through oropharyngeal inhalation. Fifty‐four mice were randomly allocated to three groups: (1) sham group, receiving saline; (2) FS group, receiving food suspension; and (3) GC group, receiving gastric contents. Each group was further divided into three subgroups according to the modeling duration (2, 4, and 8 weeks; *n* = 6 per subgroup). OA was performed once weekly, following a repeated‐exposure regimen used in prior chronic aspiration models to produce cumulative, chronic changes over time.^30^, ^31^, ^32^ Bronchoalveolar lavage fluid (BALF) and lung tissue were collected 48 h after the final exposure. Additionally, 15 mice per group were subjected to 8 weeks of modeling for survival analysis, and Kaplan–Meier curves were generated to evaluate mortality.

### 2.2 Aspiration procedures

All experiments were performed under intraperitoneal anesthesia with tribromoethanol (Beijing Jitian Biology, JT0781; 250 mg/kg). The primary modeling method was oropharyngeal aspiration (OA).^33^ Briefly, anesthetized mice were secured by the upper incisors on a 70° inclined board. The oral cavity was opened with forceps, the tongue gently extended, and the mandible stabilized to expose the pharynx and prevent swallowing. With the nostrils briefly occluded, 50 μL of the test solution was instilled into the oropharynx. This volume was selected based on the commonly used range for OA to achieve reproducible pulmonary delivery and consistent deposition.^22^, ^34^, ^35^, ^36^ Aspiration was confirmed before releasing the tongue and nostrils. Mice were then maintained in a 70° supine position for 5 min to ensure pulmonary distribution.

For comparison, intratracheal instillation (IT),^35^ intranasal inoculation (IN),^34^ and unilateral vagotomy (UV)^19^ were performed following established protocols. After each procedure, mice were kept in the 70° supine position for 5 min to promote uniform pulmonary distribution.

### 2.3 Preparation of aspiration materials

#### 2.3.1 Food suspension (FS)

Standard rodent chow (Beijing Huafukang Bioscience, 1025) was ground into fine powder, passed through a 70‐μm mesh sieve, and resuspended in normal saline at 5% (w/v) to obtain a homogeneous suspension.^29^

#### 2.3.2 Gastric contents (GC)

GC were collected from male C57BL/6 mice and pooled to minimize variability. Samples were obtained 12 h after pyloric ligation, filtered through a 70‐μm mesh to remove large particles, and stored at −80°C until use.^32^ Before administration, pH was measured and adjusted to 2–3 when necessary.

### 2.4 Bronchoalveolar lavage fluid protein concentration and cell count

Following the final aspiration, the trachea was exposed by cervical incision. Phosphate‐buffered saline (PBS) was instilled to inflate the lungs, retained for 30 s, and then withdrawn. This was repeated three times. The recovered fluid was centrifuged to separate the supernatant and cell pellet. Protein concentration in the supernatant was determined using the bicinchoninic acid (BCA) assay. The pellet was resuspended in 1 mL PBS and subjected to cell counting with a hemocytometer.^37^

### 2.5 Histopathological analysis and injury scoring

Lung tissues were fixed in 4% paraformaldehyde, embedded in paraffin, and sectioned at 4–6 μm. Sections were stained with hematoxylin and eosin (H&E) and evaluated in 20 randomly selected high‐power fields. Histopathological features, including alveolar wall thickening, inflammatory infiltration, hemorrhage, and exudation, were graded on a 0–4 scale using the Matute‐Bello system.^38^ All evaluations were independently performed by two blinded observers.

### 2.6 Immunohistochemistry and immunofluorescence analysis

Paraffin‐embedded lung sections (4 μm) were deparaffinized, rehydrated, subjected to antigen retrieval in citrate buffer (pH 6.0), and blocked with 5% bovine serum albumin. For immunohistochemistry (IHC), sections were incubated with anti–Ly‐6G antibody (Invitrogen, 14‐5931‐82, USA). For immunofluorescence (IF), primary antibodies against myeloperoxidase (MPO; R&D Systems, AF3667‐SP, USA) and citrullinated histone H3 (CitH3; Abcam, ab5103, UK) were used. Stained sections were examined by light or fluorescence microscopy. Ten high‐power fields were randomly selected, and positive cells were quantified. Positive area density was calculated using Image‐Pro Plus 6.0 (Media Cybernetics, USA).

### 2.7 Multiplex cytokines analysis

Lung tissue supernatants were collected. Inflammatory cytokines and chemokines were quantified using the ABplex Mouse Multiplex Assay Kit (ABclonal Technology, China) according to the manufacturer's instructions.

### 2.8 Micro‐CT imaging

Forty‐eight hours after aspiration, mice underwent high‐resolution micro‐CT using a SkyScan 1276 scanner (Bruker, Germany). Scanning parameters were: tube current, 200 μA; tube voltage, 85 kV; respiratory‐gated acquisition; spatial resolution, 28 μm; exposure time, 179 ms.

### 2.9 Statistical analysis

Data are presented as mean ± standard error of the mean (SEM). Group differences were assessed by one‐ or two‐way ANOVA, followed by Tukey's or Bonferroni's post hoc test. Analyses were performed using GraphPad Prism (version 10.1.2). A *p*‐value <0.05 was considered statistically significant.

## 3 RESULTS

### 3.1 OA with 5‐min vertical holding is optimal for model establishment

**FIGURE 1:**
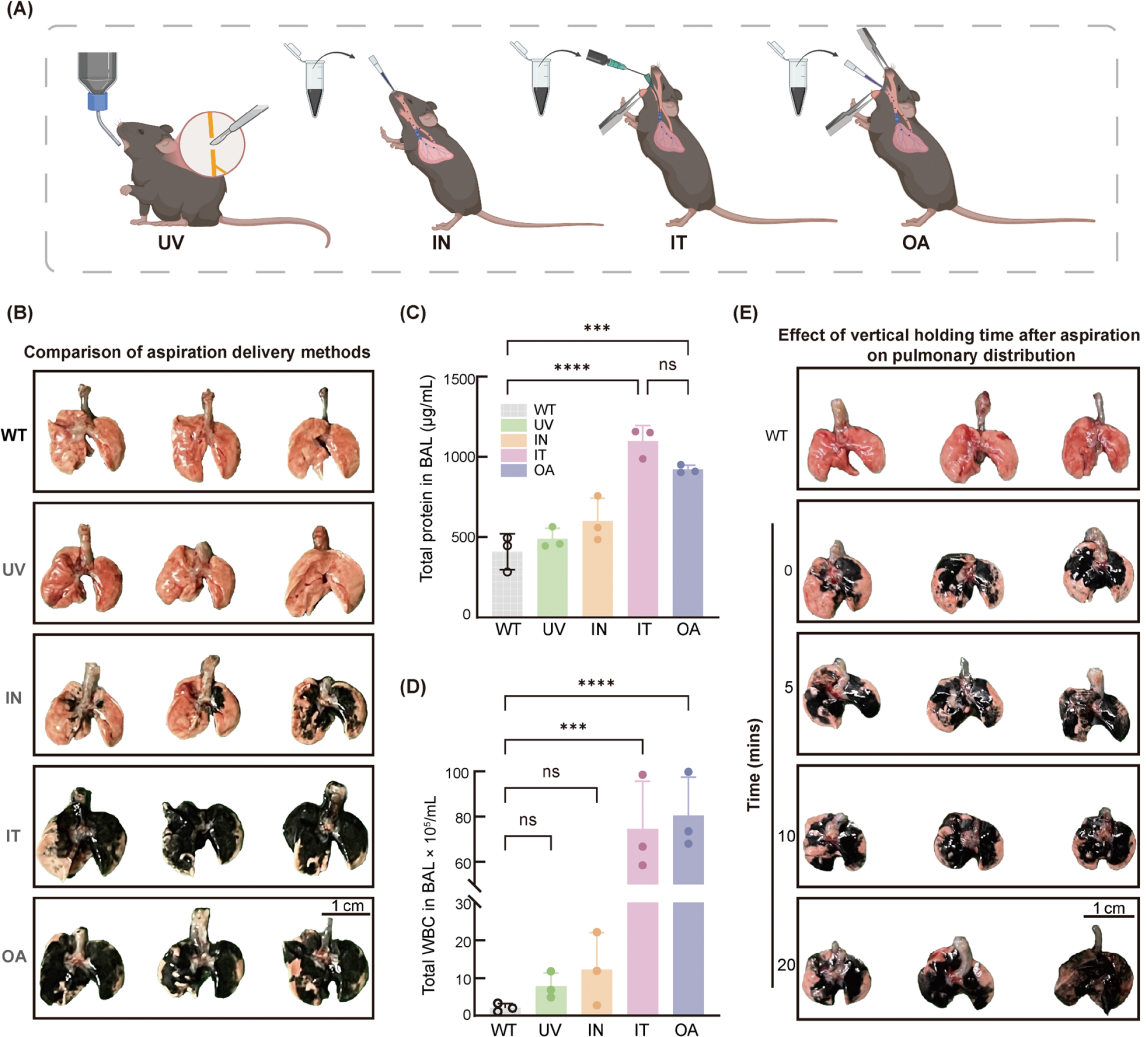
Comparison of aspiration modeling methods and optimization of oropharyngeal aspiration conditions. (A) Schematic illustration of four delivery methods: Unilateral vagotomy (UV), intranasal inoculation (IN), intratracheal instillation (IT), and oropharyngeal aspiration (OA). (B) Pulmonary distribution of India ink tracer (*n* = 3). (C, D) Total protein concentration and leukocyte counts in BALF (*n* = 3). (E) Effects of different vertical holding durations on pulmonary distribution after OA (*n* = 3). Data are presented as mean ± standard error of the mean (SEM). Each data point represents an independent animal. ***p < 0.001, ****p < 0.0001 versus control.

Four aspiration techniques were compared: UV (Figure S1), IN, IT, and OA (Figure 1A). India ink in NS was used as a tracer. Lung tissues were collected 5 min after aspiration in the IN, IT, and OA groups, and 24 h after tracer ingestion in the UV group. No deposition was observed in wild‐type lungs. In UV, only minimal staining was detected, with most tracer entering the stomach (Figure S2). IN resulted in variable and inconsistent deposition, whereas IT and OA produced uniform and widespread staining (Figure 1B). These findings confirm that IT and OA achieve efficient pulmonary delivery. Lung injury was examined 48 h after aspiration. BALF protein concentrations and leukocyte counts showed only mild, nonsignificant changes in UV and IN, but were significantly elevated in IT and OA (Figure 1C,D), indicating more severe injury. These results demonstrate that IT and OA achieved superior delivery and injury compared with UV and IN. However, IT is invasive and technically demanding, restricting its use for long‐term studies. OA, in contrast, is noninvasive, reproducible, and closer to natural aspiration, and was therefore selected as the optimal method for chronic AP modeling.

To optimize oropharyngeal aspiration efficiency, mice were held at a 70° angle after inhalation, and pulmonary distribution was assessed at 0, 5, 10, and 20 min. At 0 min, the distribution was uneven with focal aggregation, whereas uniform staining was achieved at 5 min and showed no further improvement thereafter (Figure 1E). Therefore, a 5‐min post‐aspiration vertical positioning was adopted to ensure consistent pulmonary delivery.

### 3.2 FS and GC induce lung injury

**FIGURE 2:**
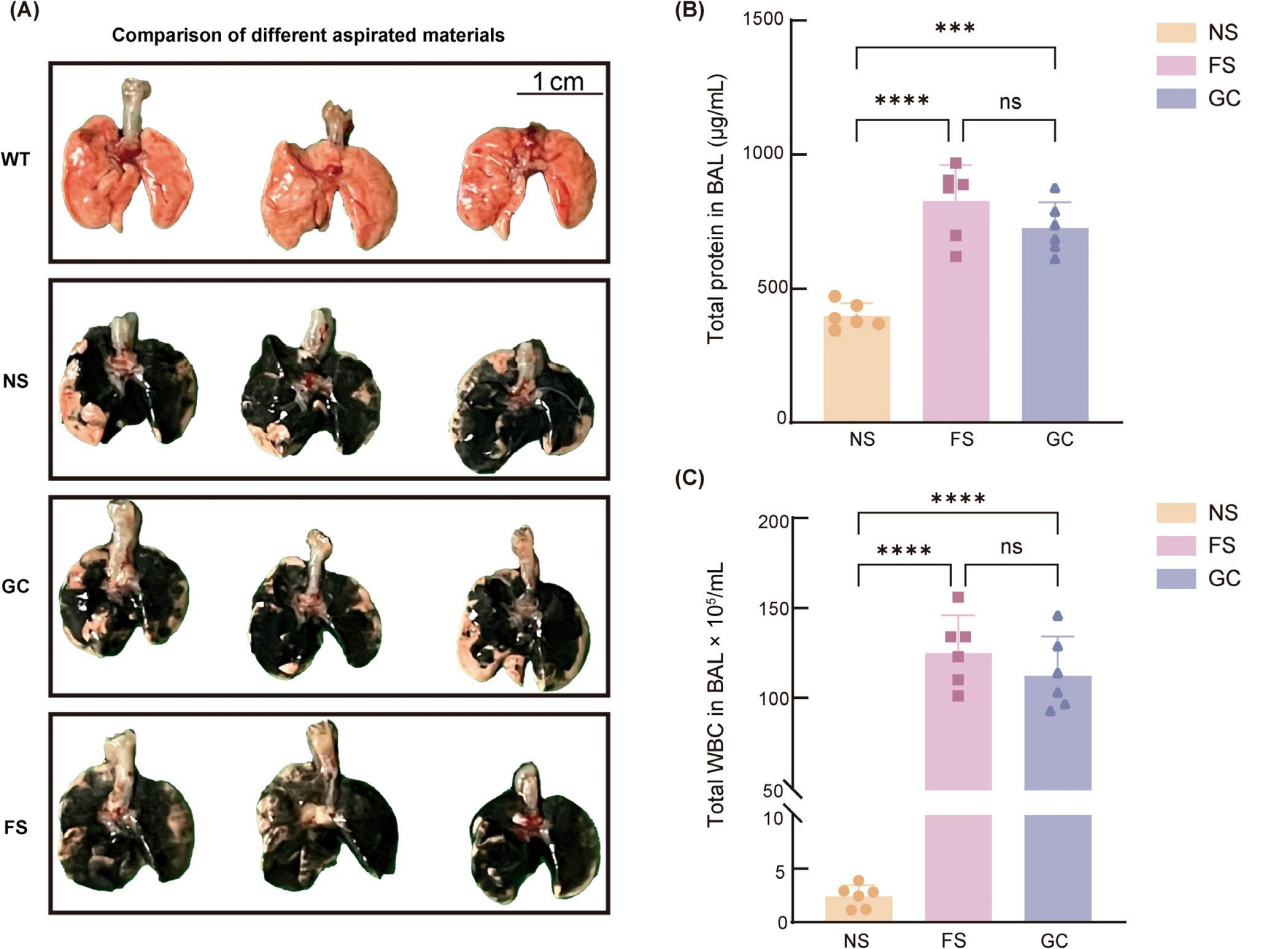
Pulmonary distribution and injury induced by different aspirated materials. (A) Pulmonary distribution of India ink mixed with NS, GC, or FS 5 min after oropharyngeal aspiration (*n* = 3). (B, C) Total protein concentration and leukocyte counts in BALF 48 h after aspiration (*n* = 6). Data are presented as mean ± standard error of the mean (SEM). Each data point represents an independent animal. ***p < 0.001, ****p < 0.0001 versus control.

To assess intrapulmonary distribution, India ink was mixed with NS, GC, or FS, and lung tissues were examined 5 min after OA. Both GC and FS showed widespread and uniform pulmonary distribution comparable to NS (Figure 2A). To further evaluate the effects of different aspirates on lung injury, BALF was collected 48 h after aspiration of NS, GC, or FS, and total protein and leukocyte counts were determined. Both BALF protein levels and leukocyte counts were significantly increased in the GC and FS groups compared with the NS group, whereas no significant difference was detected between GC and FS (Figure 2B,C). Therefore, both GC and FS delivered via OA induced comparable acute lung injury, characterized by increased alveolar–capillary permeability and inflammatory cell infiltration. These findings provided a basis for focusing subsequent experiments on chronic AP models to further differentiate the effects of FS and GC.

### 3.3 FS exacerbates alveolar–capillary barrier dysfunction

**FIGURE 3:**
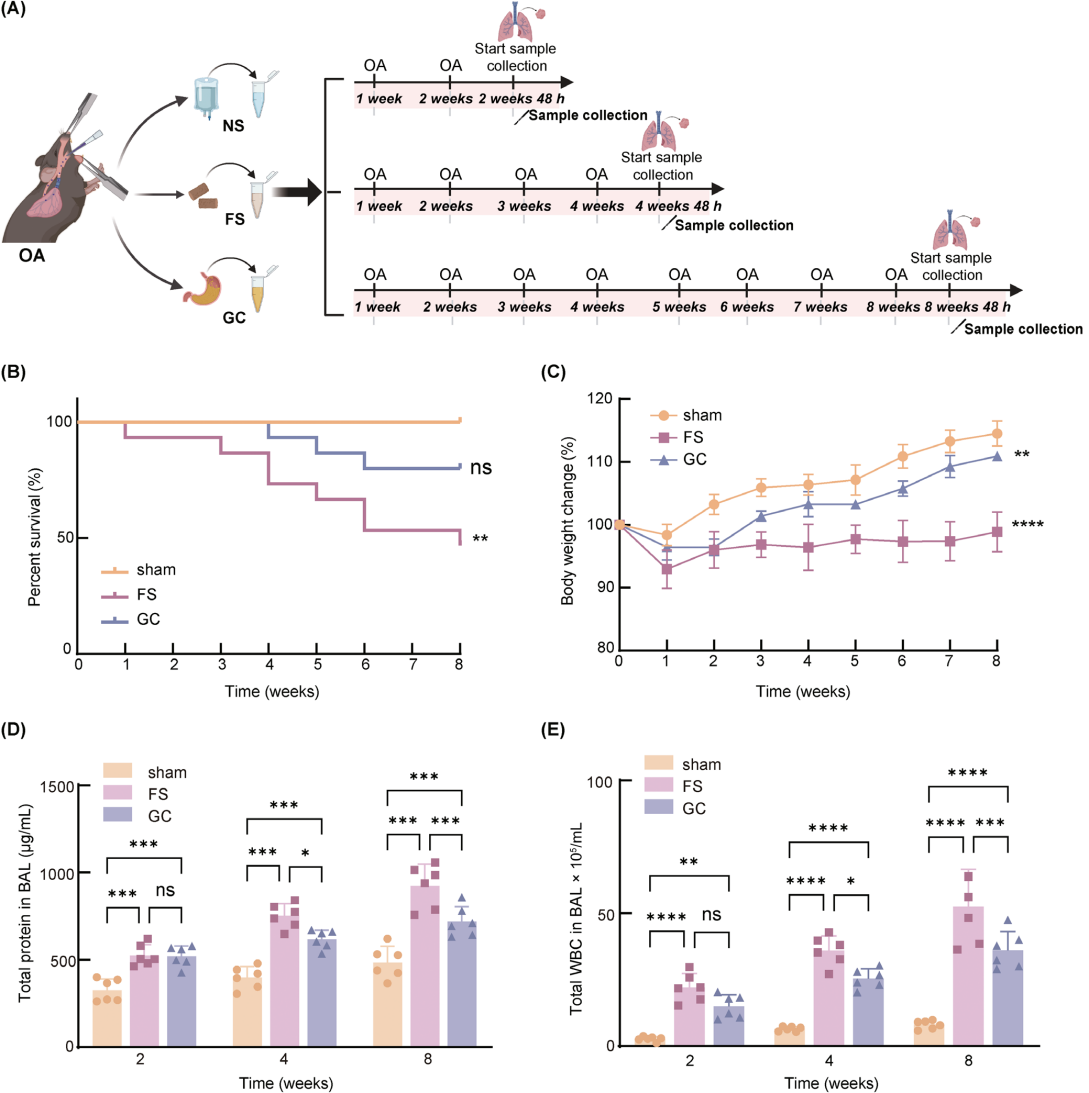
Effects of different aspirates on mouse survival, body weight, and alveolar–capillary barrier function. (A) Schematic diagram of the experimental protocol. Three experimental groups were established based on the inhaled substances: Normal saline (NS, control), food suspension (FS), and gastric contents (GC). Each group was further divided into three subgroups according to the modeling duration (2, 4, and 8 weeks; *n* = 6). Inhalation was performed once weekly, and samples were collected 48 h after the final exposure. (B) Survival curves of NS, FS, and GC groups over the 8‐week modeling period (*n* = 15). (C) Changes in body weight of NS, FS, and GC groups during the 8‐week period (*n* = 15). (D, E) Total protein concentration and leukocyte counts in BALF at each time point (*n* = 6). Data are presented as mean ± standard error of the mean (SEM). Each data point represents an independent animal. *p < 0.05, **p < 0.01, ***p < 0.001, ****p < 0.0001 versus control.

To establish a chronic AP mouse model, NS was used as the control, and FS and GC served as experimental aspirates. Observations were performed at weeks 2, 4, and 8 (Figure 3A). Survival analysis showed a significant decline in FS‐exposed mice, whereas survival in GC was comparable to sham (Figure 3B). Body weight increased progressively in the sham and GC groups but remained lower in FS (Figure 3C). To further assess alveolar–capillary barrier permeability, BALF was collected at weeks 2, 4, and 8. Both GC and FS groups showed increased protein concentrations and leukocyte counts at all time points compared with the sham group, with injury severity progressively increasing over time (Figure 3D,E). Notably, FS consistently exhibited higher levels than GC at weeks 4 and 8, indicating more severe and sustained alveolar–capillary barrier damage.

### 3.4 FS induces severe histopathological injury

**FIGURE 4:**
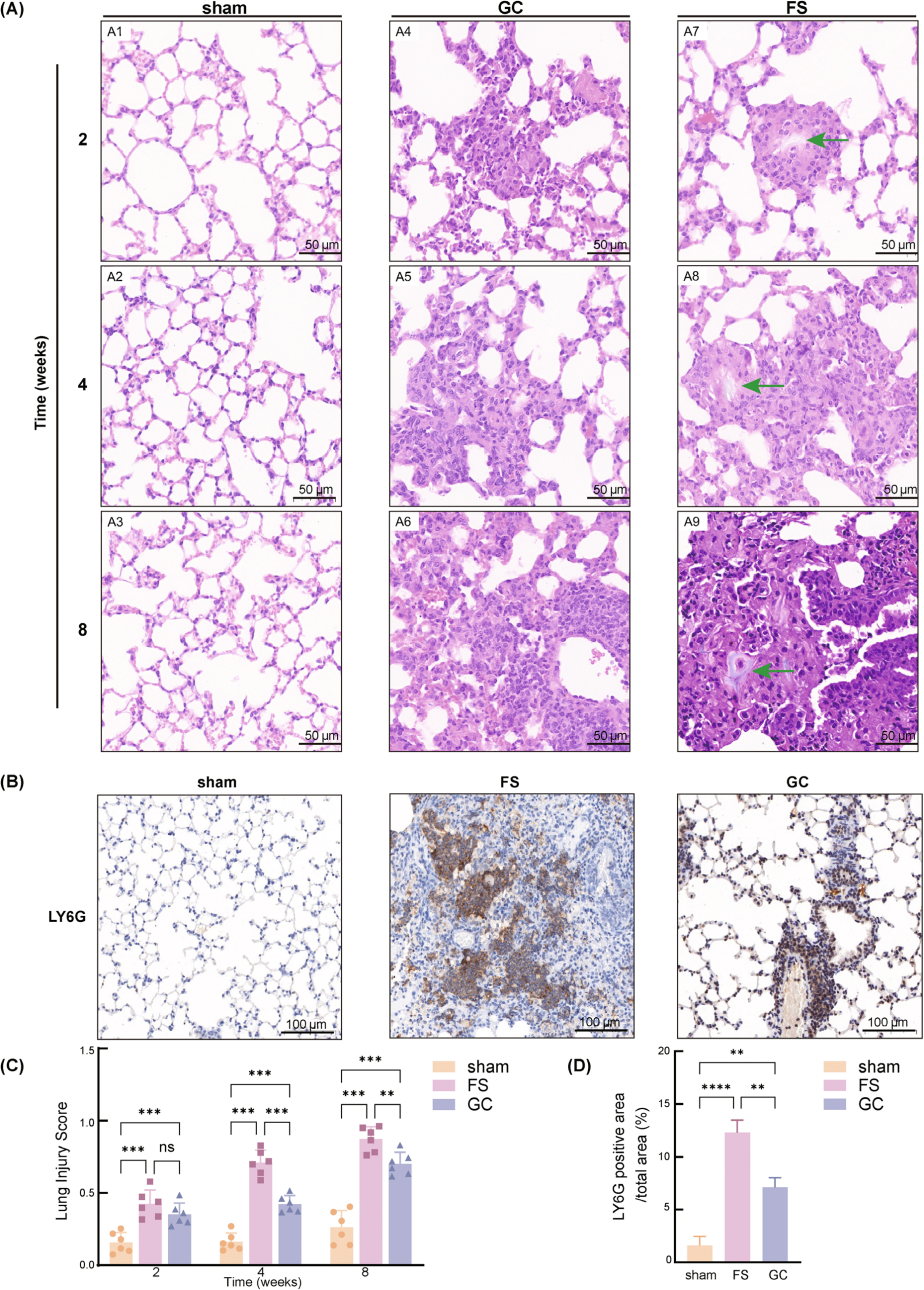
Histopathological changes in mouse lungs induced by food suspension and gastric contents. (A) H&E staining of lung sections at 2, 4, and 8 weeks in each group (green arrows indicate food particles; scale bar, 50 μm). (B) Immunohistochemical staining of neutrophil marker Ly6G in lung sections at 8 weeks (scale bar, 100 μm). (C) Lung injury scores (*n* = 6). (D) Quantitative analysis of Ly6G‐positive area (*n* = 3). Data are presented as mean ± standard error of the mean (SEM). Each data point represents an independent animal. **p < 0.01, ***p < 0.001, ****p < 0.0001 versus control.

Histopathological changes were assessed in FS‐ and GC‐exposed lungs at weeks 2, 4, and 8. H&E staining showed preserved alveolar architecture without inflammatory infiltrates in sham mice (Figure 4A1–A3). At week 2, GC lungs displayed mild septal widening with minimal eosinophilic infiltration. By weeks 4 and 8, eosinophilic infiltration and obliterative bronchiolitis were evident, with no detectable foreign material deposition (Figure 4A4–A6). In contrast, FS lungs showed food particle deposition with periparticle eosinophilic infiltration at week 2. By week 8, FS lungs exhibited marked septal widening around deposited particles, alveolar collapse, and severe obstructive bronchiolitis (Figure 4A7–A9). Lung injury scores were higher in FS than in GC and increased over time, consistent with the histological findings (Figure 4C). Notably, obliterative bronchiolitis with retained food particles, a key feature of human AP that is rarely reproduced in rodent models, occurred only in FS.^13^, ^39^, ^40^

At week 8, Ly6G staining revealed increased neutrophil infiltration in FS and GC lungs compared with sham (Figure 4B,D), with FS showing the strongest signal. Overall, neutrophil infiltration was more pronounced in FS than in GC at week 8.

### 3.5 FS induces a strong inflammatory response

**FIGURE 5:**
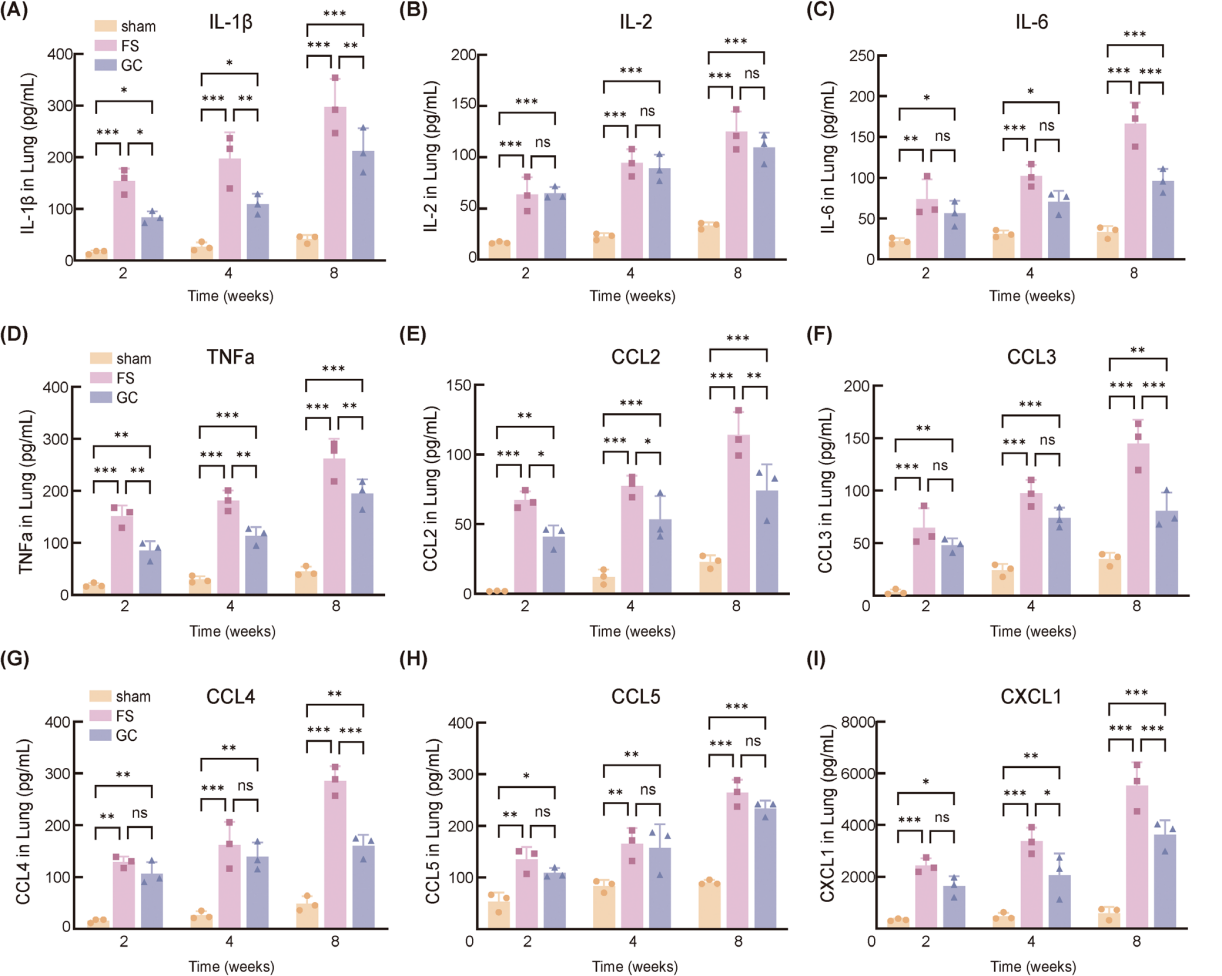
Effects of different aspirated materials on inflammatory mediator levels in mouse lungs. (A–D) Concentrations of IL‐1β, IL‐2, IL‐6, and TNF‐α in lung tissue at 2, 4, and 8 weeks after aspiration (*n* = 3). (E–I) Concentrations of CCL2, CCL3, CCL4, CCL5, and CXCL1 in lung tissue at 2, 4, and 8 weeks after aspiration (*n* = 3). Data are presented as mean ± standard error of the mean (SEM). Each data point represents an independent animal. *p < 0.05, **p < 0.01, ***p < 0.001, ****p < 0.0001 versus control.

Lung tissues were analyzed at weeks 2, 4, and 8 to assess cytokine and chemokine expression in chronic AP (Figure 5A–I). Compared with sham, both FS and GC showed increased inflammatory mediator levels as early as week 2, which increased further at later time points and reached higher levels by week 8. Overall, FS and GC showed similar temporal patterns in cytokine levels. Compared with GC, FS generally showed greater increases over time, indicating that aspirate composition is a key determinant of inflammatory response intensity.

### 3.6 Micro‐CT reveals gravity‐dependent pulmonary consolidation

**FIGURE 6:**
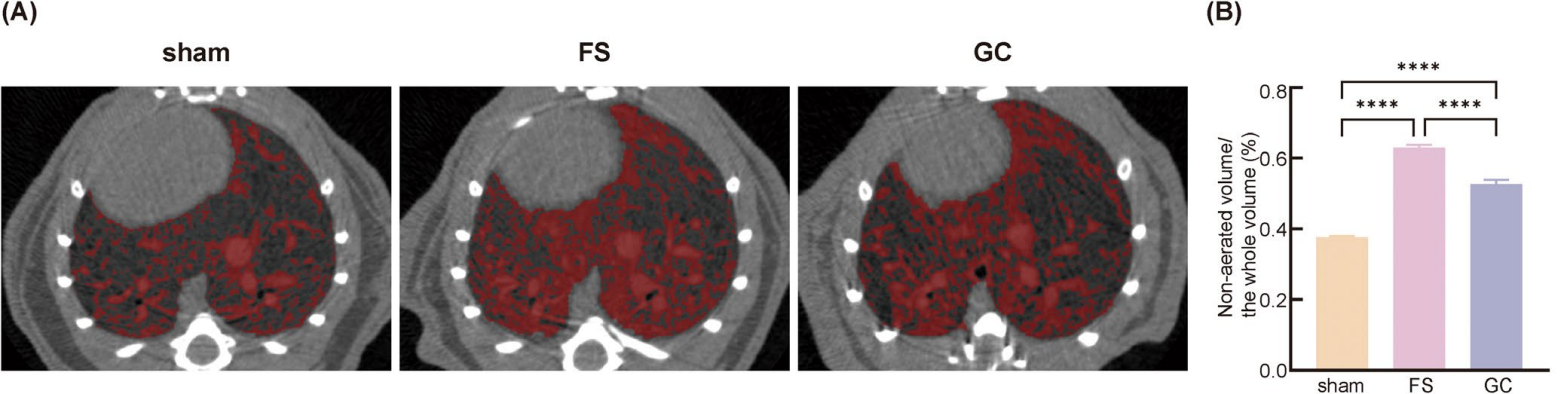
Micro‐CT evaluation of ventilation abnormalities and gravity‐dependent consolidation in mouse lungs. (A) Representative micro‐CT images showing abnormal ventilation regions (red) in the lungs of sham, FS, and GC groups. (B) Quantitative analysis of hypoventilated and non‐ventilated regions as a proportion of total lung volume (*n* = 3). Data are presented as mean ± standard error of the mean (SEM). Each data point represents an independent animal. ****p < 0.0001 versus control.

Micro‐CT was performed at week 8, when injury was most severe, to assess lung structure and ventilation. Compared with sham, both FS and GC showed impaired ventilation. FS additionally exhibited gravity‐dependent consolidation, mainly in the basal segments of the lower lobes (Figure 6A). Quantitative analysis confirmed that FS had a significantly higher proportion of hypoventilated lung volume than sham and GC (Figure 6B). Together, these findings indicate that FS delivered via OA induces a gravity‐dependent consolidation pattern consistent with radiographic features commonly observed in human AP.^41^, ^42^

### 3.7 Transcriptomic analysis identifies activation of the NETs pathway

**FIGURE 7:**
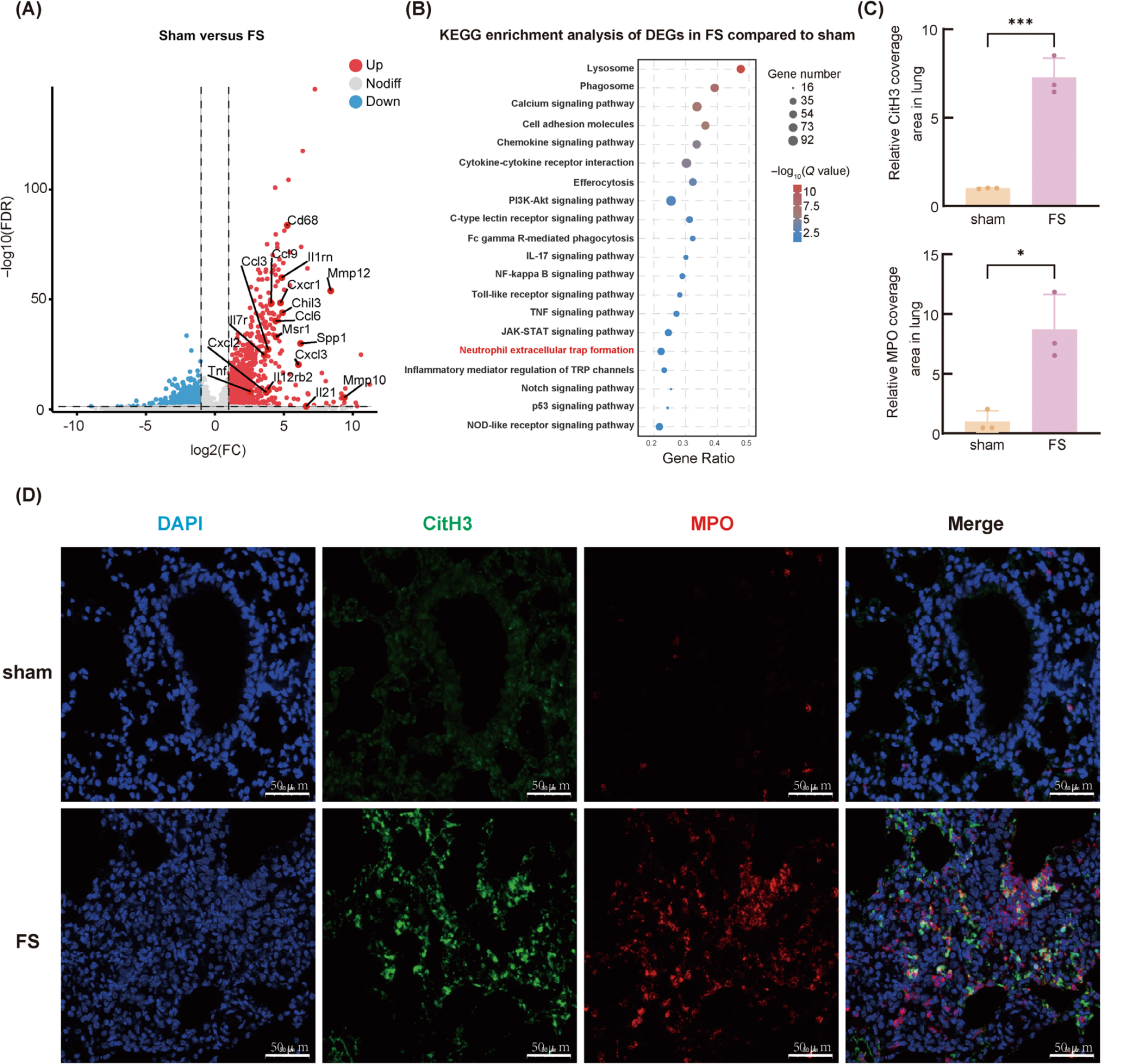
Transcriptomic and immunofluorescence analyses indicate NETs formation in mouse lungs. (A) Volcano plot showing differentially expressed genes in lung tissue from FS and sham groups (*n* = 5). (B) KEGG pathway enrichment analysis of differentially expressed genes. (C) Quantification of CitH3 and MPO levels in lung tissue from FS and sham groups (*n* = 3). (D) Immunofluorescence staining showing colocalization of CitH3 (green) and MPO (red) in lung tissue, consistent with NETs formation (scale bar, 50 μm). Data are presented as mean ± standard error of the mean (SEM). Each data point represents an independent animal. *p < 0.05, ***p < 0.001 versus control.

RNA sequencing was performed on FS‐exposed lungs at week 8, the time point most representative of chronic AP. Volcano plot analysis showed marked transcriptional alterations compared with sham (Figure 7A). FS lungs exhibited strong upregulation of proinflammatory, leukocyte recruitment, and extracellular matrix remodeling genes, including Il17f, Cxcl2, and Mmp10. Differential expression of TNF receptor family members (Tnfrsf1b, Tnfrsf23, Tnfrsf26) further indicated persistent inflammatory activation. KEGG pathway analysis (Figure 7B) identified enrichment of multiple inflammation‐ and immunity‐related pathways, such as IL‐17, NF‐κB, and PI3K–Akt signaling. Notably, the NETs formation pathway was significantly enriched. To validate NETs activation, immunofluorescence showed clear colocalization of CitH3 and MPO in FS lungs at week 8 (Figure 7C,D), consistent with active NETs formation. These findings demonstrate NETs formation in chronic AP and suggest their potential involvement in disease progression.

## 4 DISCUSSION

In this study, we established a reproducible mouse model of chronic AP using a noninvasive OA approach. Weekly FS aspiration disrupted the alveolar–capillary barrier, induced obliterative bronchiolitis with retained food particles, and triggered sustained inflammatory responses. It also produced gravity‐dependent consolidation on micro‐CT and activated NET‐associated pathways. Collectively, these findings indicate that the model captures key pathological features of chronic AP and provides a relevant platform for mechanistic studies.

Clinical aspiration in AP is often subtle and recurrent, with long‐term microaspiration leading to diffuse bronchiolitis.^11^, ^43^ Previous animal models of AP include acute models generated by direct airway delivery and chronic models established through repeated IT/IN exposure.^17^, ^29^, ^30^, ^31^ Although informative for understanding AP‐associated inflammation and tissue remodeling, these approaches do not fully capture physiological aspiration, and procedural consistency is easily compromised during long‐term, repeated aspiration. We therefore used OA to establish stable chronic phenotypes under more physiological conditions, providing a foundation for subsequent mechanistic comparisons across aspirate compositions.

The composition of aspirated material is a key driver of AP related lung injury.^11^, ^39^ Liquid stimulation models represented by hydrochloric acid or LPS have distinct pathological emphases. Hydrochloric acid primarily causes barrier disruption with increased permeability, whereas LPS predominantly drives inflammatory cell infiltration with alveolar edema or hemorrhage.^17^, ^19^ However, these single component liquid stimuli do not reproduce clinically common composite aspiration events such as FS and GC.^26^, ^27^ Barrier function can recover rapidly after acute aspiration,^17^, ^19^ whereas chronic repeated aspiration produces cumulative injury and progressive remodeling. In chronic aspiration studies, repeated GC exposure is associated with interstitial and perivascular inflammation and multinucleated giant cell reactions.^30^ In contrast, particle containing FS more readily promotes particle retention with granulomatous inflammation and structural destruction.^29^ In our model, FS showed greater particle retention with progression to granulomatous inflammation and obstructive bronchiolitis than GC. Micro CT in FS exposed lungs showed gravity dependent consolidation in the basal segments of the lower lobes. Overall, the FS induced imaging and histopathological phenotype was consistent with reports in older adults with chronic AP.^13^, ^39^, ^41^, ^44^

During prolonged repeated aspiration, FS induced higher inflammatory mediator levels, indicating stronger immune activation in response to particulate food, potentially driven by mechanical injury and delayed clearance.^45^, ^46^, ^47^, ^48^ Compared with GC based models that mainly emphasize macrophage dominant inflammation and fibrosis,^30^ we observed more prominent neutrophil infiltration with increased NETs formation, suggesting a neutrophil oriented inflammatory profile in chronic AP. This pattern also echoes reports from acute aspiration models describing PANoptosis related regulated cell death implicated in early tissue injury.^17^ Given the association between excessive NETs formation and worse outcomes in pneumonia and acute respiratory distress syndrome,^49^, ^50^, ^51^ future studies may employ PAD4 deficiency or pharmacological inhibition of NETs formation to clarify the role of NETs in chronic AP lung injury.

This study has limitations. First, only young male mice were used, whereas AP predominantly affects older adults. Previous studies indicate that anesthesia increases aspiration susceptibility in aged animals, suggesting that age related impairment of airway protective reflexes may heighten aspiration risk.^52^, ^53^ Second, infectious factors were not included, although AP is often associated with bacterial infection.^54^ Nevertheless, by minimizing pathogen related confounding, this model more directly captures aspiration induced barrier disruption, small airway injury, and radiographic phenotypes. This design provides experimental evidence that disentangles the respective contributions of aspirate induced injury and secondary infection to disease progression.

## 5 CONCLUSION

In summary, we established a mouse model that faithfully reproduces the key pathological and imaging features of human chronic AP. The model is characterized by progressive alveolar–capillary barrier dysfunction, obstructive bronchiolitis, and gravity‐dependent consolidation. By addressing the limitations of acute aspiration models, this study highlights the crucial role of aspirate composition in chronic disease progression. It also provides a valuable platform for mechanistic research and therapeutic exploration.

## AUTHOR CONTRIBUTIONS

**Qianwen Li:** Data curation; formal analysis; investigation; methodology; visualization; writing – original draft; writing – review and editing. **Fansen Lin:** Data curation; investigation; methodology. **Hongzhi Gao:** Resources; supervision. **Yun Zhao:** Project administration; supervision; writing – review and editing. **Yuqi Liu:** Conceptualization; funding acquisition; project administration; resources; supervision; writing – review and editing.

## FUNDING INFORMATION

This study was supported by grants from the Major Projects of Fujian Provincial Health Commission (2021ZQNZD008).

## CONFLICT OF INTEREST STATEMENT

The authors declare no conflicts of interest.

## ETHICS STATEMENT

All animal experiments were conducted in accordance with the principles of laboratory animal welfare and the relevant regulations of the Fujian Medical University Laboratory Animal Ethics Committee, with ethical approval number IACUC‐FJMU2025‐Y‐0346.

## Supporting information


**Data S1:** Supplementary figures and tables.
