# Supplementary material for: Establishment of a chronic aspiration pneumonia mouse model using oropharyngeal aspiration of food suspension
Source: Animal Model Exp Med. 2026 Mar 24;9(5):1002–13. doi: 10.1002/ame2.70197 (PMC13331564; doi:10.1002/ame2.70197)
Supplement: Supplementary file 1 — Data S1: Supplementary figures and tables. [file AME2-9-1002-s001.docx]

**Supplementary Material**


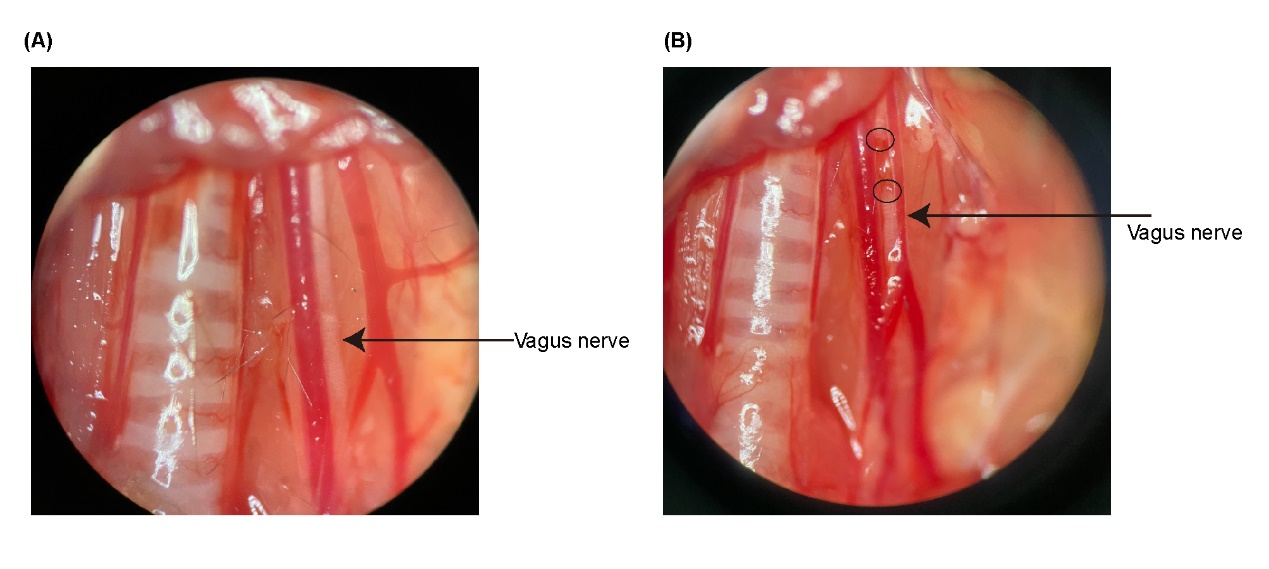


**Figure S1. Unilateral vagotomy procedure.** **(A)** Exposure of the unilateral vagus nerve. **(B)** Transection of the unilateral vagus nerve.

**
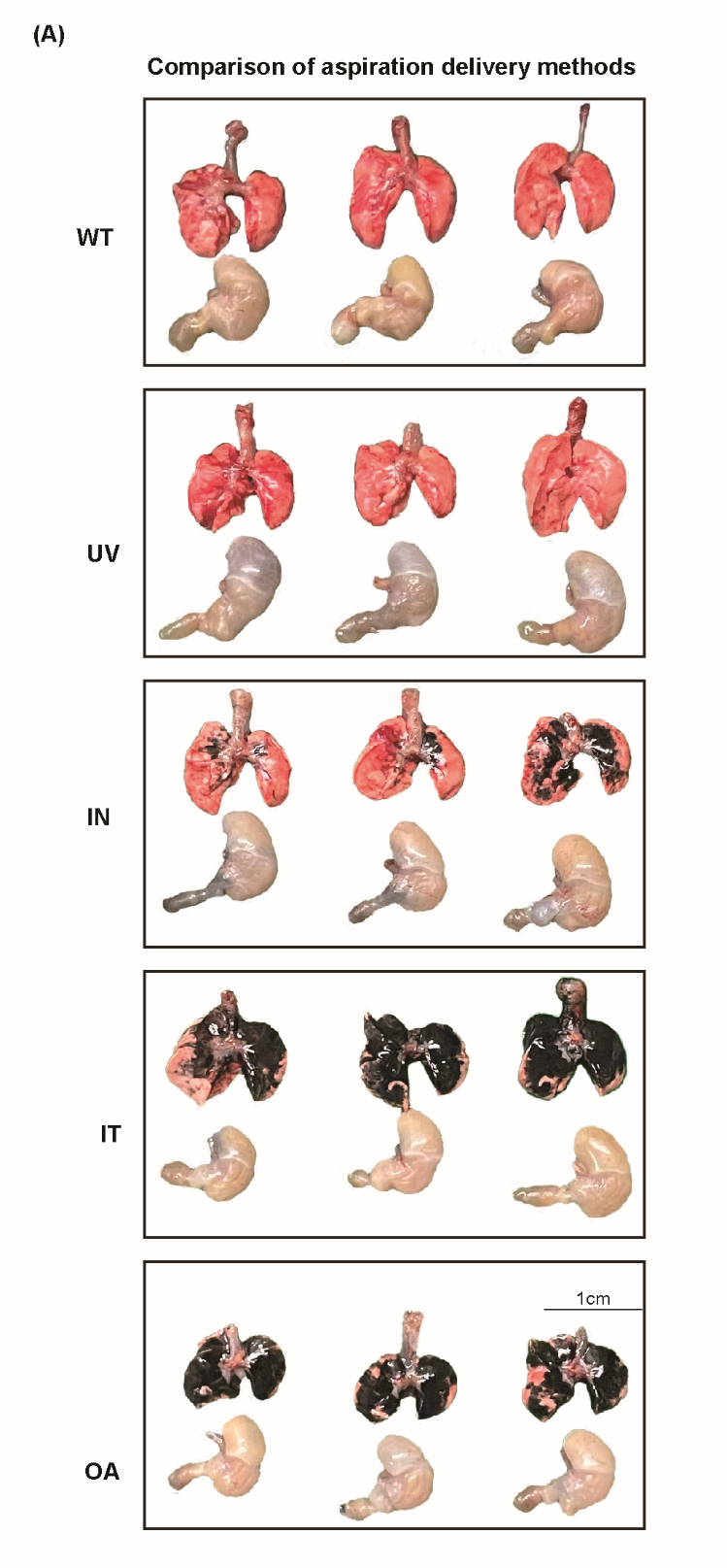
**

**Figure S2. Comparison of inhalation modeling methods. (A)** Distribution of India ink tracer in the stomach and lungs (n = 3).
